# Ursolic acid mitigates hepatic ischemia-reperfusion injury by regulating the ALOX12/12(S)-HETE and PTGES/prostaglandin E2 axis via arachidonic acid metabolism pathway

**DOI:** 10.3389/fphar.2026.1781014

**Published:** 2026-04-07

**Authors:** Wen Hou, Jiansen Lu, Hao Pan, Huanyu Wang, Xuequan Feng, Hongsheng Liu

**Affiliations:** 1 NHC Key Laboratory of Critical Care Medicine, Tianjin Organ Transplant Research Center, Tianjin First Central Hospital, Tianjin, China; 2 Department of Hepatobiliary Surgery, Tianjin First Central Hospital, Tianjin, China; 3 School of Medicine, Nankai University, Tianjin, China; 4 Neurosurgery Department, Tianjin First Central Hospital, Tianjin, China

**Keywords:** hepatic ischemia and reperfusion injury, uroslic acid, ALOX12/12(S)-HETE, arachidonic acid metabolism pathway, non-target metabolomics, PTGES/prostaglandin E2

## Abstract

**Introduction:**

Hepatic ischemia-reperfusion injury (HIRI) is a critical contributor to poor prognosis after hepatobiliary surgery, yet effective therapeutic strategies remain limited. Ursolic acid (UA), a natural pentacyclic triterpenoid, has shown potential hepatoprotective properties, but its specific mechanism in alleviating HIRI remains unclear. This study aimed to investigate the therapeutic effect of UA on HIRI and elucidate its underlying molecular mechanisms, addressing the clinical need for targeted interventions.

**Methods:**

A murine HIRI model was established, and UA was administered to assess its impact on liver function. Serum levels of aspartate aminotransferase (AST) and alanine aminotransferase (ALT) were measured to evaluate hepatoprotection. Integrated network pharmacology and non-targeted metabolomics were employed to identify key pathways and metabolites involved in UA-mediated protection. Molecular docking was used to predict interactions between UA and target proteins. Experimental validation included assessment of the ALOX12/12(S)-HETE and PTGES/prostaglandin E2 axes. A cellular thermal shift assay (CETSA) was performed to confirm direct binding between UA and the PTGES protein.

**Results:**

UA administration significantly reduced serum AST and ALT levels in HIRI mice, confirming its protective role against liver injury. Integrated multi-omics analysis revealed the arachidonic acid metabolic pathway as a central hub for UA’s protective effects. Key metabolites (prostaglandin H2 and prostaglandin E2) were markedly downregulated by UA, and UA downregulated the ALOX12/12(S)-HETE and PTGES/prostaglandin E2 axes. CETSA confirmed that UA directly binds to the PTGES protein, supporting its role as a target of UA.

**Conclusion:**

UA attenuates HIRI primarily by modulating the arachidonic acid metabolic pathway and inhibiting the ALOX12 and PTGES signaling axes. These findings highlight UA as a promising therapeutic agent for HIRI, with potential for clinical translation to improve outcomes in hepatobiliary surgery.

## Graphical Abstract

**Figure:**
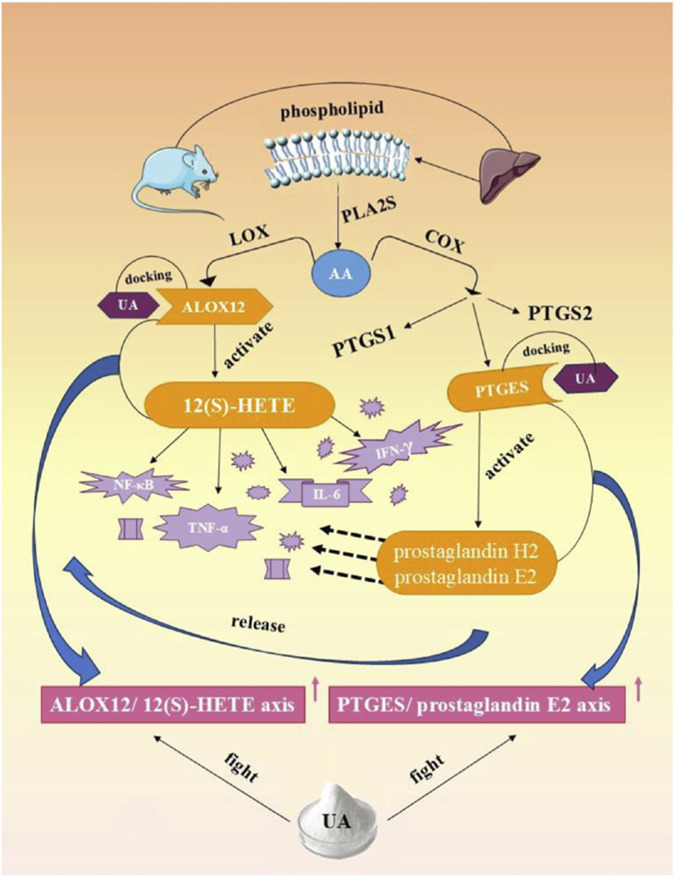

## 1 Introduction

Hepatic ischemia-reperfusion injury (HIRI) refers to the pathological process in which liver tissue sustains additional damage upon the restoration of blood flow following a period of ischemia. It is an unavoidable complication in hepatic surgeries such as liver transplantation and hepatic resection, and represents a major cause of postoperative liver dysfunction and failure (Du et al., 2022; Liu et al., 2024). The underlying mechanisms of HIRI are complex, but primarily involve oxidative stress and robust inflammatory responses (Zhang W. et al., 2023; George et al., 2024). Ischemia itself induces cellular damage due to deficiency in oxygen and nutrients. However, upon reperfusion, the sudden influx of oxygen leads to excessive generation of reactive oxygen species (ROS), which in turn disrupts cellular membranes, organelles, and DNA, and activates inflammatory pathways that further amplify tissue injury (Alva et al., 2018; Tang et al., 2022; Gracia-Sancho et al., 2021). Despite its clinical significance, the molecular pathways of HIRI are not fully elucidated, and effective therapeutic or preventive measures are still lacking, underscoring the urgency of identifying new drug targets and pharmacological strategies.

Network pharmacology has emerged as a transformative discipline that synthesizes systems biology, computational science, and big data analytics to investigate drug-disease interactions from a holistic standpoint. Centered on the concept of “network targets”-key nodes within biological networks whose modulation can shift disease phenotypes—this approach provides a powerful framework for deciphering complex mechanisms and predicting therapeutic outcomes, particularly for multi-target agents and traditional medicines (Hu et al., 2024; Nogales et al., 2022). Complementing this paradigm, metabolomics—first introduced by Professor Nicholson in 1999—enables the comprehensive profiling of endogenous low-molecular-weight metabolites (<1,000 Da), thereby capturing systemic metabolic responses to pathological or pharmacological perturbations. Its inherently holistic and dynamic perspective aligns well with the multi-component, multi-target nature of herbal medicines, positioning it as an apt tool for elucidating their mechanisms of action (Li and Zhang, 2013; Li et al., 2011; Shi et al., 2024; Zhang XY. et al., 2023).

Ursolic acid (UA) is a naturally occurring pentacyclic triterpenoid abundant in medicinal plants such as apples, hawthorn, and Prunella vulgaris. It possesses a wide range of pharmacological properties, including anti-inflammatory, antioxidant, hepatoprotective, and anti-tumor activities (Nguyen et al., 2021; Lei et al., 2023; Xu et al., 2024). Importantly, toxicological studies have confirmed its safety; no significant toxicity was observed in rats even at doses as high as 1,000 mg/kg/day (Geerlofs et al., 2020).

Previous work from our team established that dogwood in which UA is a principal bioactive component, mitigates HIRI in mice through regulation of the PTGS2 pathway (Hou et al., 2024). The present study evaluates the specific role of UA in murine HIRI and delineates its mechanism of action using an integrated approach that combines network pharmacology with non-targeted metabolomics. This multi-omics strategy allows a comprehensive exploration of the pharmacological network through which UA exerts its protective effects, offering valuable insights for clinical translation and drug discovery.

## 2 Materials and methods

### 2.1 Animal and drug treatment

Male C57BL/6 mice (5–6 weeks old, 20–22 g) were obtained from the Institute of Experimental Animals, Chinese Academy of Medical Sciences (SCXK Beijing 2014-0004). Mice were housed under SPF conditions (23 °C ± 1.5 °C, 45% ± 10% humidity) with free access to food and water. Ursolic acid (UA, SU8082, ≥98% purity, Beijing Solaibao Technology Co., Ltd.) was dissolved in 0.5% sodium carboxymethyl cellulose and administered via gavage at doses of 10 mg/kg (low dose) or 30 mg/kg (high dose) body weight.

### 2.2 Animal experiment

Thirty mice were randomly divided into five groups (n = 6/group): Sham, Sham + high-dose UA (Sham + HUA), HIRI model, HIRI + low-dose UA (HIRI + LUA), and HIRI + high-dose UA (HIRI + HUA). UA-treated groups received daily intragastric administration for 7 days; control groups received vehicle. One hour after the last dose, the IRI model was established in accordance with the method previously described (Yu et al., 2025). After a 12-h fasting period, during which the mice had free access to water, anesthesia was induced via pentobarbital sodium (40 mg/kg, i.p.). Once fully anesthetized, the mice were secured on a dedicated animal operating table, and the abdominal skin was disinfected using iodophor. A midline laparotomy was then performed to expose the abdominal cavity. Subsequently, the intestines were carefully wrapped with sterile, moistened gauze and gently retracted to one side, providing unobstructed access to the hepatic portal region. The left and median liver lobes, accounting for approximately 70% of the total liver volume, were clamped using a non-invasive arterial clip to induce ischemia. After maintaining ischemia for 1 h, the clip was removed to restore blood flow, marking the onset of reperfusion. Following reperfusion, the intestines were meticulously returned to their original anatomical position. The abdominal layers were then closed sequentially, ensuring proper surgical closure. Postoperatively, the mice were placed on a heating pad to maintain body temperature and ensure recovery under optimal conditions. After 6 h of reperfusion, the animals were euthanized, and both serum and liver tissue samples were collected for subsequent analysis.

### 2.3 Serum biochemical analysis

Serum was separated from blood samples and levels of AST and ALT were measured using an automatic biochemical analyzer (BS-240VET, Mindray Shenzhen).

### 2.4 Liver histopathological examination

Liver tissues were fixed in 10% formalin, embedded in paraffin, sectioned at 5 μm, and stained with hematoxylin and eosin (H&E). Histopathological changes were examined under light microscopy.

### 2.5 Network pharmacology analysis

Firstly, we employed multiple databases and tools, including TCMSP (http://tcmspw.com./tcmsp.php), CancerHSP (https://old.tcmsp-e.com/CancerHSP.php), ChEMBL (https://www.ebi.ac.uk/chembl/), Pharm Mapper (http://lialb-ecust.cn/pharmmapper/), and Swiss Target Prediction (http://www.swisstargetprediction.ch/), to predict the target genes corresponding to UA. The species was set to *Homo sapiens*. After obtaining the target genes, we merged them and removed duplicates. Subsequently, these genes were standardized and transformed on the UniProt platform (http://www.uniprot.org/). Ultimately, UniProt IDs were utilized to represent the target genes, thereby establishing a database for UA active ingredient targets. Next, we used “hepatic ischemia - reperfusion injury” as the keyword in the GeneCards (http://www.genecards.org/), OMIM (Online Mendelian Inheritance in Man), and NCBI (https://www.ncbi.nlm.nih.gov/) databases to predict the target genes associated with HIRI. These target genes were then corrected and transformed on the UniProt service platform and finally represented by UniProt IDs. The intersection target genes of UA and HIRI were collected. Cytoscape3.7.2 software was employed to construct a visual network of the UA - HIRI interaction.

### 2.6 Pathway enrichment analysis

The common target genes of UA and HIRI obtained above were input the PathDIP (http://ophid.utoronto.ca/pathD) and species set to *H. sapiens*, *P < 0.01* and *FDR < 0.05* as screening conditions. Meanwhile, the common target gene was input into Cytoscape3.7.2 plugin ClueGO for pathway analysis, and the screening condition was set that the species was human and *P < 0.05*. Through analyzing the pathway data of common target genes in the two databases, we screened the target pathway and then discussed the mechanism of UA intervention in HIRI.

### 2.7 Untargeted metabolomics

Liver samples (n = 6 per group) from Sham, HIRI, and HIRI + UA (high-dose) groups were analyzed by untargeted metabolomics (Novo Zhiyuan Biotechnology, Beijing). Metabolic pathways were identified using MetaboAnalyst 5.0.

### 2.8 Multi-omics integration

Common pathways from network pharmacology and metabolomics were intersected. MetScape (Cytoscape plugin) was used to identify hub genes associated with key metabolites within these pathways.

### 2.9 Molecular docking

3D structures of hub target proteins were downloaded from PDB, and the UA structure was obtained from PubChem. Molecular docking was performed using the DockThor online server to evaluate binding affinity.

### 2.10 Immunohistochemistry

Liver sections underwent antigen retrieval, peroxidase blocking, and incubation with primary antibodies against PTGES and ALOX12. HRP-conjugated secondary antibodies and DAB chromogen were used for detection. Nuclei were counterstained with hematoxylin.

### 2.11 Western blot

Proteins were extracted from liver tissue, quantified using a BCA kit, separated by SDS-PAGE, and transferred to PVDF membranes. Membranes were probed with anti-ALOX12 and anti-PTGES antibodies (1:500), followed by species-matched secondary antibodies. Protein bands were visualized using chemiluminescence and quantified with ImageJ.

### 2.12 Cellular thermal shift assay (CETSA)

Cellular thermal shift assay (CESTA) could be used to verify the binding of cells to potential protein targets (Luo et al., 2022). AML12 hepatocytes were treated with 10 μM UA or 0.1% DMSO for 2 h. Cells were heated at different temperatures (37 °C–81 °C), lysed, and centrifuged. Supernatants were analyzed by western blot to assess protein thermal stability upon UA binding.

### 2.13 Data analysis

Data are presented as mean ± SEM. Differences between groups were analyzed using one-way ANOVA or Student’s t-test in GraphPad Prism 8.0. A p-value <0.05 was considered statistically significant.

## 3 Result

### 3.1 UA ameliorates liver injury induced by hepatic IRI in mice

**FIGURE 1:**
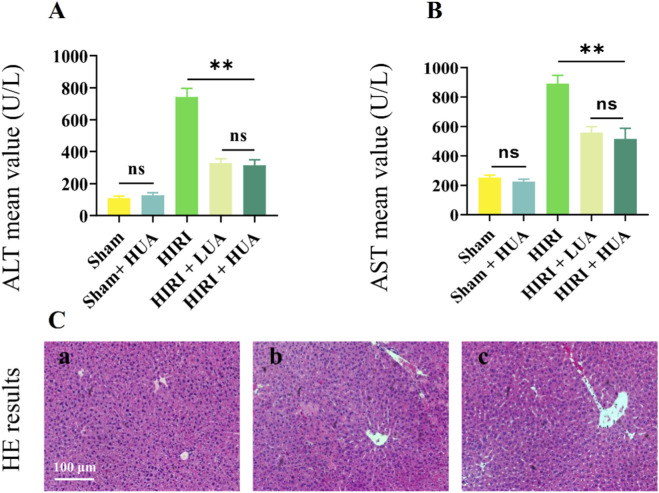
UA pretreatment ameliorates liver injury after IRI. **(A,B)** The levels of ALT and AST. **(C)** HE staining of mice liver in sham **(a)** IRI **(b)** and UA pretreatment **(c)** group (n = 6).

The chemical structure of ursolic acid (C_30_H_48_O_3_, molecular weight 456.71 g/mol) is well-documented in PubChem (Supplementary Figures 1A, B). To evaluate the therapeutic effect of UA on liver IRI, mice were pretreated with UA for 7 days before the IRI surgery. Both low-dose and high-dose UA treatments significantly reduced ALT and AST levels after hepatic IRI, indicating a protective effect against IRI. UA did not alter ALT or AST levels in sham-operated mice, demonstrating its safety in normal liver tissue (Figures 1A,B). Given that no significant dose-dependent effect was observed between the two UA treatment groups, we selected the low-dose UA group for subsequent experiments. Histological examination via H&E staining revealed that UA treatment alleviated hepatocellular swelling, necrosis, steatosis, and inflammatory infiltration compared to the untreated group. This suggests that UA can mitigate IRI-induced liver pathology (Figure 1C).

### 3.2 Screening and identification of potential therapeutic targets

**FIGURE 2:**
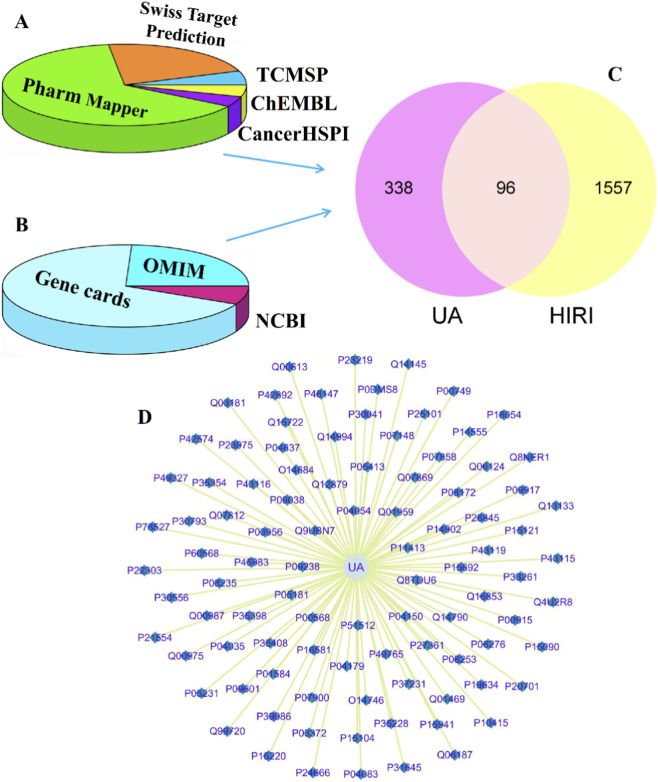
Network of UA target genes. **(A)** Target genes of UA from the TCMSP, CancerHSP, ChEMBL, PharmMapper and SwissTargetPrediction databases. **(B)** Target genes of HIRI from the GeneCards, NCBI and OMIM databases. **(C)** Venn diagram (pink represents UA, yellow represents HIRI). **(D)** Network diagram depicting the interrelationships between UA and HIRI by Cytoscape.

Cross-validation from five databases, including TCMSP, CancerHSP, ChEMBL, PharmMapper, and SwissTargetPrediction, yielded 465 potential targets of UA (Figure 2A). Using “hepatic ischemia-reperfusion injury” as the keyword, searches in GeneCards, NCBI, and OMIM identified 2,213 HIRI-related genes (Figure 2B). After ID standardization and deduplication, 434 UA targets and 1,653 HIRI targets were retained, with 96 overlapping targets between them (Figure 2C). A network diagram was constructed using Cytoscape to visualize interactions, where UA was represented as circular nodes, HIRI-related genes as triangular nodes, and their associations as connecting lines (Figure 2D). This analysis systematically outlines the molecular interactions between UA and HIRI, laying the foundation for further mechanistic studies.

**FIGURE 3:**
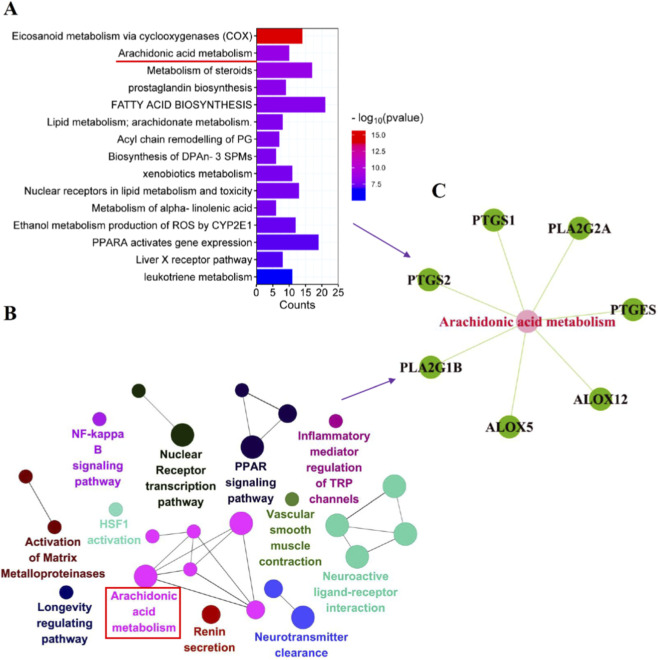
Pathway analysis of UA-HIRI common target genes. **(A,B)** Pathways in PathDIP and ClueGO. **(C)** Arachidonic acid metabolic pathway enriched by both databases.

The 96 common targets between UA and HIRI were analyzed using the PathDIP and ClueGO databases. PathDIP identified 136 significantly enriched pathways, while ClueGO yielded 67 entries (Figures 3A,B). The arachidonic acid metabolism pathway was prominently highlighted in both analyses. Further intersection of target genes within this pathway from both databases identified seven key genes: ALOX12, ALOX5, PLA2G1B, PLA2G2A, PTGES, PTGS1, and PTGS2 (Figure 3C).

### 3.3 Metabolomics analysis

A total of 18 mice were included in this study and allocated into three experimental groups: Sham, IRI, and IRI + HUA (n = 6/group), with interposed QC samples to ensure data robustness. PCA demonstrated that the three groups exhibited distinct clustering and were well-separated along the principal component axis in both ESI positive and negative modes, indicative of profound differences in their metabolomic profiles (Supplementary Figures 2A, B). To further explore inter-group differences, a supervised PLS-DA model was constructed. The 7-fold cross-validation parameters met acceptable criteria (R^2^ > Q^2^, Q^2^ regression intercept <0), excluding the risk of overfitting and confirming the model’s suitability for screening differential metabolites (Supplementary Figures 2C–J). These results validated the robustness of the model and its applicability for identifying differential metabolites.

**FIGURE 4:**
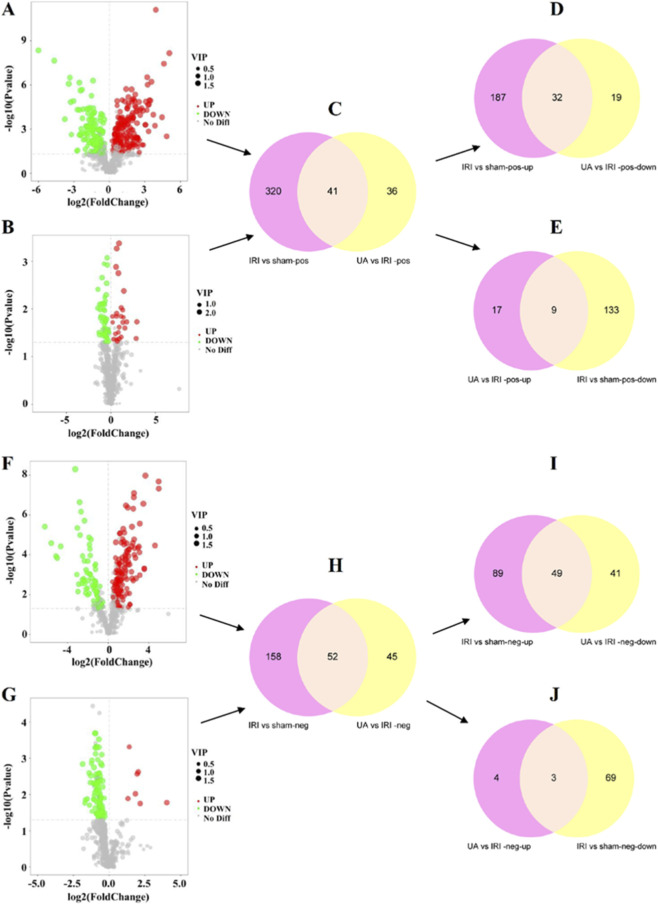
Differential metabolites analysis in positive mode **(A–E)** and in negative mode **(F–J)**.

Differential metabolites were identified based on the criteria of VIP >1.0, |FC| > 1.0, and *P* < 0.05. In positive ion mode, 322 metabolites were differentially expressed in the IRI group compared to the Sham group, of which 191 were upregulated and 131 were downregulated (Figure 4A). Compared to the IRI group, the UA group showed 77 differential metabolites, including 26 upregulated and 51 downregulated (Figure 4B). Intersection analysis revealed 41 common differential metabolites between the two comparisons (IRI vs. Sham and UA vs. IRI) (Figures 4C–E). In negative ion mode, 210 differential metabolites were identified in the IRI group compared to the Sham group (138 upregulated and 72 downregulated, Figure 4F). The UA intervention group exhibited 97 differential metabolites compared to the IRI group (7 upregulated and 90 downregulated, Figure 4G), with 52 common differential metabolites between the two comparisons (Figures 4H–J). Hierarchical clustering analysis (HCA) further revealed the expression patterns of the differential metabolites: most differential metabolites were highly expressed in the IRI group (highlighted in red) and lowly expressed in the Sham group (highlighted in dark blue), while the UA treatment group showed intermediate expression levels (highlighted in light blue), with significant differences in expression patterns among the groups (Supplementary Figures 3A, B). Correlation analysis based on Pearson coefficients clarified the interactions among metabolites, with both positive correlations (highlighted in red) and negative correlations (highlighted in blue) clearly presented (Supplementary Figures 3C, D). Notably, the number of negative correlations detected in positive ion mode was significantly higher than that in negative ion mode.

### 3.4 Multi-omics integration of differential metabolite pathways

MetaboAnalyst 5.0 was used to perform pathway enrichment and target association analysis on the differential metabolites identified in positive ion mode (41 metabolites) and negative ion mode (52 metabolites). Using a threshold of *P* < 0.05, 7 and 8 significantly enriched pathways were identified in positive and negative ion modes, respectively.

**FIGURE 5:**
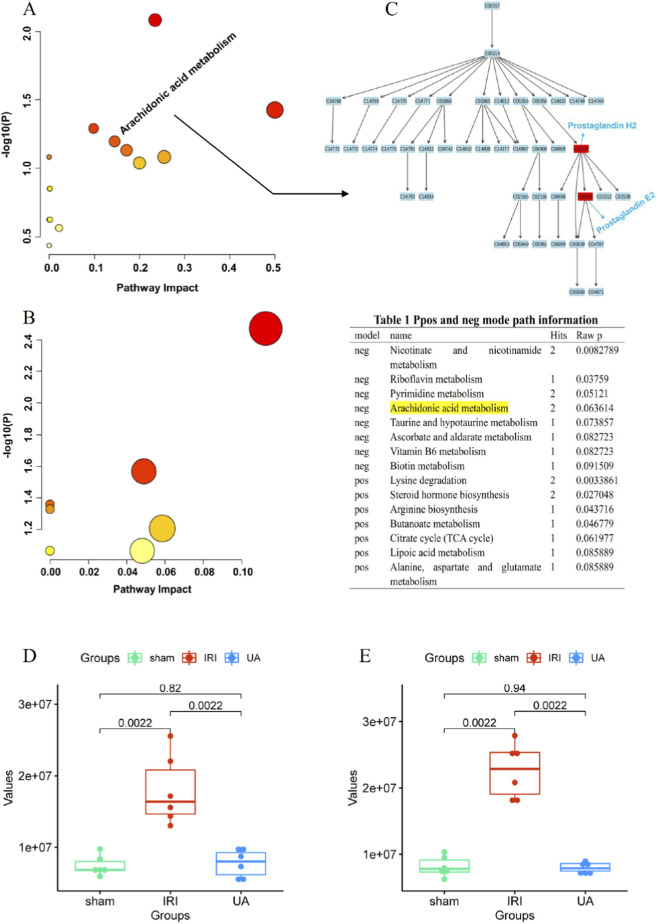
Differential metabolites analysis. **(A,B)** Enriched metabolic pathways of network pharmacology analysis under pos and neg conditions. **(C)** Key metabolites in the Arachidonic acid metabolism pathway. **(D)** The level of Prostaglandin H2. **(E)** the level of Prostaglandin E2.

**FIGURE 6:**
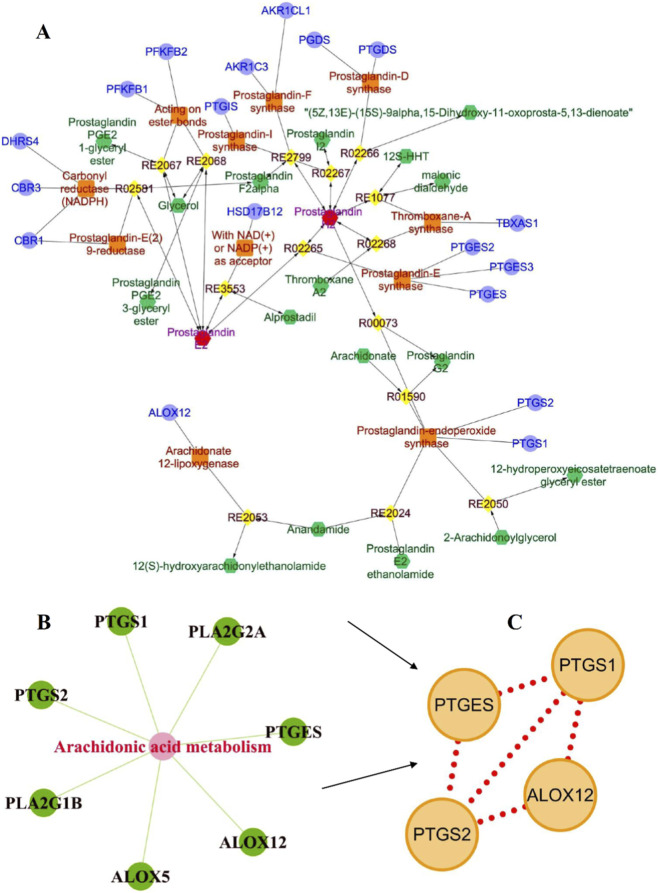
Acquisition of hub target genes. **(A)** Network in MetScape of Cytoscape using KEGG codes C00427 and C00584 (corresponding to prostaglandin H2 and E2). The pink nodes represent the input metabolites, the green ones represent the compounds, the orange ones represent the enzymes, the lavender ones represent the genes, and the yellow ones represent the reactions. **(B)** Target genes corresponding to prostaglandin H2 and E2. **(C)** Core target genes.

Notably, the arachidonic acid metabolism pathway, which was significantly enriched in negative ion mode, perfectly aligned with the core pathway identified in our prior network pharmacology analysis (Figures 5A,B). This cross-omics convergence directly validates on this pathway as the central mechanism by which UA alleviates HIRI. Focusing on this pathway, and integrating analyses of metabolite abundance and functional associations, we identified prostaglandin H2 (PGH2) and prostaglandin E2 (PGE2) as key effector metabolites (Figure 5C). Quantitative validation revealed that compared to the Sham group, the levels of both PGH2 and PGE2 were significantly elevated in the HIRI group. After UA intervention, their abundances were significantly suppressed (Figures 5D,E). This dynamic change—“elevated upon injury and suppressed by UA intervention”—directly confirms that UA exerts its anti-HIRI effects by regulating key metabolites in this pathway. To decipher the upstream and downstream regulatory relationships between metabolites and genes, a target gene network for PGH2 and PGE2 was constructed using the Cytoscape plugin MetScape, screening 18 directly related target genes (Figure 6A). These were cross-analyzed with the “7 key targets in the arachidonic acid metabolism pathway” identified from the earlier network pharmacology screening, ultimately pinpointing the core regulatory genes: ALOX12, PTGES, PTGS1, and PTGS2 (Figures 6B,C). These genes serve as core functional nodes within the pathway, directly mediating metabolite synthesis and signal transduction, and thus represent key molecular targets for UA against HIRI.

### 3.5 UA downregulates the ALOX12/12(S)-HETE and PTGES/Prostaglandin E2 axes in HIRI

To clarify the interaction between UA and the core targets, molecular docking of UA with ALOX12, PTGES, PTGS1, and PTGS2 was performed using the DockThor online tool. The results showed that UA can form stable complexes with all four proteins via hydrogen bonds: forming 2 hydrogen bonds with ALOX12 (PDB ID: 3RDE), 3 with PTGES (PDB ID: 5T36), 2 with PTGS1 (PDB ID: 6Y3C), and 1 with PTGS2 (PDB ID: 5F19) (Supplementary Figures 4A–H). Regarding docking scores, ALOX12 (−7.886) and PTGES (−7.419) exhibited stronger binding affinity. Based on the number of hydrogen bonds (reflecting binding stability) and functional relevance (core nodes in the arachidonic acid metabolism pathway), ALOX12 and PTGES were selected as the primary molecular mediators of UA against HIRI for subsequent validation.

**FIGURE 7:**
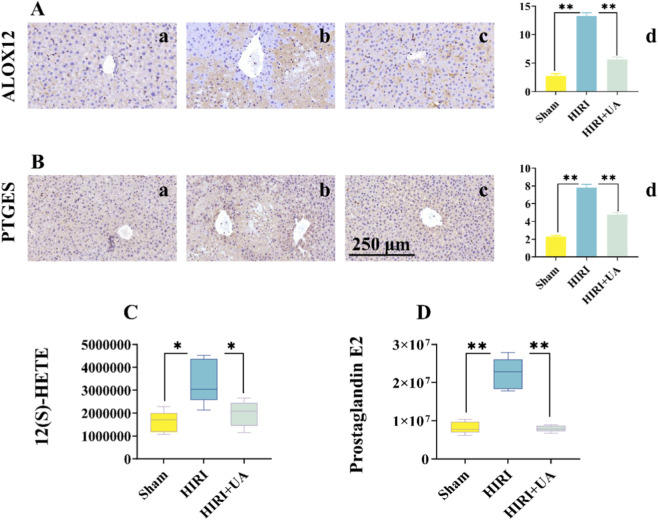
UA Downregulates the ALOX12/12(S)-HETE and PTGES/Prostaglandin E2 Axes in HIRI. **(A)** Immunohistochemistry of ALOX expressions in sham **(a)** IRI **(b)** and UA **(c)** group. (n = 3). **(B)** Immunohistochemistry of PTGES expressions in sham **(a)** IRI **(b)** and UA **(c)** group. (n = 3). **(C,D)** Metabolomics analysis of the metabolites 12(S)-HETE **(C)** and Prostaglandin E2 **(D)**.

Immunohistochemistry demonstrated a significant increase in ALOX12 and PTGES protein expression in the HIRI group relative to the Sham group, which was attenuated upon UA treatment (Figures 7A,B). This pattern was paralleled by metabolomic data: the levels of 12(S)-HETE and prostaglandin E2 (PGE2), the respective downstream metabolites of ALOX12 and PTGES, were markedly elevated in the HIRI group and effectively normalized by UA (Figures 7C,D). These results confirm that UA alleviates HIRI by inhibiting the ALOX12/12(S)-HETE and PTGES/PGE2 signaling axes.

### 3.6 UA stabilizes PTGES through direct binding

**FIGURE 8:**
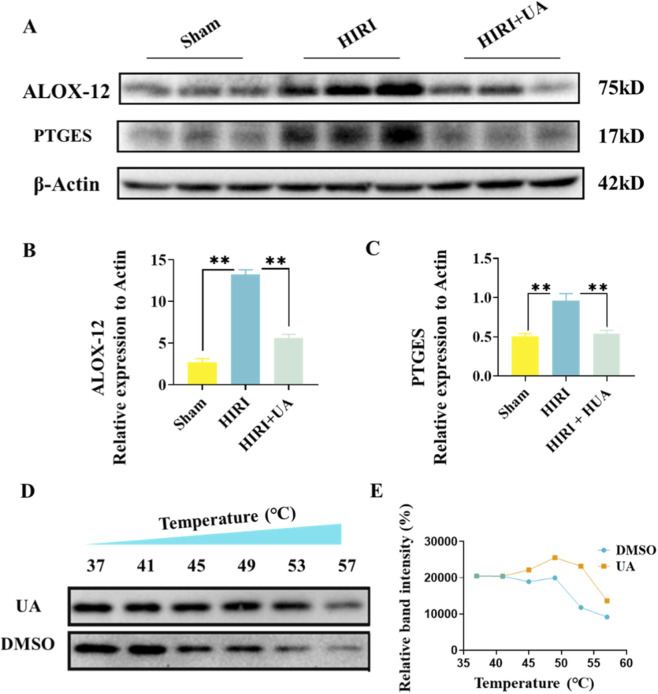
UA stabilizes PTGES through direct binding. **(A)** Western blot of ALOX12 and PTGES in different groups (n = 3). **(B,C)** Relative expression of ALOX12 **(B)** and PTGES **(C)**. **(D)** Western blot of CETSA results of UA treatment in AML12 cell line. **(E)** Denaturation temperature curve.

Western blot analysis revealed that compared to the sham group, the protein expression of ALOX12 and PTGES in liver tissues of the HIRI model group was significantly increased. However, after UA intervention, the expression of both proteins was markedly reduced (Figures 8A–C), which was consistent with findings in the UA-treated group shown in Figures 7A,B. These findings further indicate that UA may exert its protective effects by downregulating the expression of ALOX12 and PTGES, thereby mitigating the progression of HIRI. In AML12 cells not treated with UA, the melting curve of PTGES began to shift at 45 °C. However, after UA treatment, the denaturation temperature of PTGES increased to 57 °C, indicating enhanced thermal stability due to the direct binding of UA to the active site of PTGES (Figures 8D,E). These results demonstrate that UA forms a stable complex with PTGES, providing mechanistic insights into its role in alleviating HIRI.

## 4 Discussion

HIRI critically contributes to postoperative liver dysfunction and remains a significant clinical challenge in hepatobiliary surgery (Liu and Man, 2023). Identifying effective therapeutic strategies is therefore of paramount importance. UA, a natural compound present in various medicinal plants and fruits, has emerged as a promising candidate due to its notable biological activities and safety profile. Previous investigations by Piao et al. (Piao et al., 2021) outlined the temporal dynamics of HIRI, wherein metabolic dysregulation dominates the initial 0-3 h post-ischemia, followed by Kupffer cell activation and inflammatory cytokine release that peaks around 6 h. Informed by this pathophysiology, our study focused on the 6-h reperfusion mark to elucidate the protective mechanisms of UA. Supporting its pharmacological potential, Geerlofs et al. (Geerlofs et al., 2020) confirmed that UA exhibits no toxicity in rats even at high doses (1,000 mg/kg/day), affirming its safety for further exploration. In the present study, two doses of UA (10 mg/kg and 30 mg/kg) were evaluated in a murine HIRI model. Both doses significantly reduced serum AST and ALT levels compared to the HIRI group (**P < 0.01), indicating potent hepatoprotection. This study preliminarily confirmed the protective effect of UA against HIRI. However, there was no significant difference in efficacy between the 10 mg/kg and 30 mg/kg dose groups (P > 0.05), suggesting that subsequent studies need to set a wider dose gradient and increase the sample size to systematically plot the dose-effect curve, thereby clarifying the minimum effective dose of UA, the efficacy plateau, and the optimal dosing regimen.

Using an integrated multi-omics strategy combining network pharmacology and non-targeted metabolomics, we identified the arachidonic acid metabolism pathway as central to UA’s mechanism. Key components of this pathway-ALOX12, PTGES, and their metabolites 12(S)-HETE, prostaglandin H2, and prostaglandin E2, were consistently dysregulated in HIRI and significantly normalized by UA treatment. Specifically, levels of these markers were elevated in the HIRI group (**P < 0.01 or *P < 0.05) and markedly suppressed following UA administration, confirming the involvement of the ALOX12/12(S)-HETE and PTGES/prostaglandin E2 axes. The arachidonic acid pathway mediates inflammation and cell damage through several enzymatic branches (Zhong et al., 2023; Zhang et al., 2021). Once liberated by phospholipase A2 (PLA2), arachidonic acid is metabolized via cyclooxygenase (COX), lipoxygenase (LOX), and cytochrome P450 (CYP450) enzymes into various eicosanoids (Zhang et al., 2018; L et al., 2024). Our data indicate that during HIRI, the LOX pathway, particularly ALOX12, drives production of 12(S)-HETE, which promotes hepatocyte injury and promotes the expression of pro-inflammatory cytokines including TNF-α, IL-6, and IFN-γ. Concurrently, the COX pathway elevates PTGS1, PTGS2, and PTGES, resulting in excessive synthesis of prostaglandin H2 and E2, further amplifying inflammatory damage (Tanaka et al., 2024; Wang et al., 2025; Zhang et al., 2022). UA appears to mitigate HIRI by targeting these two key axes. The broader implications of these pathways are underscored by their roles in other diseases. Geng et al. (Geng et al., 2023) reported that the PTGES/PGE2 axis modulates glycolysis and chemosensitivity in colorectal cancer. Wang et al. (Chen et al., 2020; Qian et al., 2025) correlated this axis with tumor initiation and metastasis in lung cancer models. Similarly, Yang et al. (Yang et al., 2019) and Zhang et al. (Zhang et al., 2021; Zhang et al., 2018) demonstrated the critical involvement of ALOX12/12(S)-HETE in liver IRI and myocardial injury, while Gao et al. (Gao et al., 2020) and Chen et al. (Chen et al., 2020) implicated it in leukemia and lung cancer chemoresistance, respectively. The consistent upregulation of these pathways across multiple pathologies reinforces their biological significance and aligns with our findings in HIRI.

Molecular docking analysis is a reliable and efficient analytical approach to predict the binding affinity between molecules (Qian et al., 2025). The docking score of UA and ALOX12 or PTGES is similar, but we focus on PTGES for CETSA test. That is because PTGES is the rate-limiting enzyme for PGE2 synthesis and is located at a key downstream node in this inflammatory cascade. Inhibiting PTGES can directly and effectively block the massive production of PGE2, thereby alleviating inflammatory damage. In future work, we will supplement the CETSA experiment of UA with ALOX12 to comprehensively validate the regulatory effect of UA on this signaling axis.

This study preliminarily revealed the mechanism by which UA alleviates HIRI through the ALOX12/12(S)-HETE and PTGES/PGE_2_ axes in the arachidonic acid metabolic pathway via network pharmacology and non-targeted metabolomic analysis. However, this study still has several limitations that are worthy of in-depth exploration in subsequent research. Firstly, the causal roles of ALOX12 and PTGES in this process have not been verified at the gene level, such as loss-of-function experiments using small interfering RNA (siRNA) or CRISPR gene knockout technology. Secondly, the study only observed a single time point of 6 h after reperfusion, failing to reflect the dynamic changes of related pathways over time. Thirdly, although an animal model was used, it was not verified in combination with an *in vitro* hypoxia/reoxygenation (H/R) model, and there was also a lack of absolute quantitative analysis of metabolites (such as liquid chromatography-tandem mass spectrometry multiple reaction monitoring technology, LC-MS/MS/MRM) and cross-validation using methods such as enzyme-linked immunosorbent assay (ELISA). Fourthly, the inhibitory effect of UA on the target enzymes was mainly inferred from the indirect changes at the protein or metabolite level, and has not been confirmed by direct enzyme activity assays. In addition, advanced bioinformatics methods such as protein-protein interaction (PPI) network analysis and molecular dynamics simulation were not included in the analysis scope of this study.

To address the above deficiencies, subsequent research will be carried out in the following aspects: First, use siRNA or CRISPR gene knockout technology to verify the causal association between ALOX12 and PTGES in the protective effect of UA at the genetic level. Second, expand the sampling range of reperfusion time points to comprehensively analyze the dynamic change characteristics of related pathways. Third, introduce an *in vitro* hypoxia/reoxygenation (H/R) model to supplement animal experimental data, and combine absolute quantification methods such as LC-MS/MS/MRM and ELISA technology for cross-validation to improve the accuracy and reproducibility of detection results. Fourth, conduct direct enzyme activity assays to confirm the inhibitory effect of UA on ALOX12 and PTGES. In addition, computational biology methods such as PPI network topology analysis and molecular dynamics simulation will also be used to further elucidate the molecular mechanism of UA at the systematic level. Through the above research, we will more comprehensively clarify the protective effect of UA on HIRI, providing a more solid theoretical basis for its potential use as a therapeutic drug.

## 5 Conclusion

Integrated network pharmacology and non-targeted metabolomics revealed that ursolic acid (UA) alleviates hepatic ischemia-reperfusion injury (HIRI) probably by targeting ALOX12 and PTGES, leading to suppression of the ALOX12/12(S)-HETE and PTGES/prostaglandin E2 axes. These findings highlight UA as a promising therapeutic candidate for mitigating HIRI, offering a novel strategy for clinical intervention in ischemia-reperfusion-related liver damage.

## Data Availability

The original contributions presented in the study are included in the article/Supplementary Material, further inquiries can be directed to the corresponding authors.
