# Supplementary material for: Ursolic acid mitigates hepatic ischemia-reperfusion injury by regulating the ALOX12/12(S)-HETE and PTGES/prostaglandin E2 axis via arachidonic acid metabolism pathway
Source: Front Pharmacol. 2026 Apr 7;17:1781014. doi: 10.3389/fphar.2026.1781014 (PMC13096074; doi:10.3389/fphar.2026.1781014)
Supplement: Supplementary file 1 [file Supplementaryfile1.docx]

**Supplementary**

Supplementary Fig .1


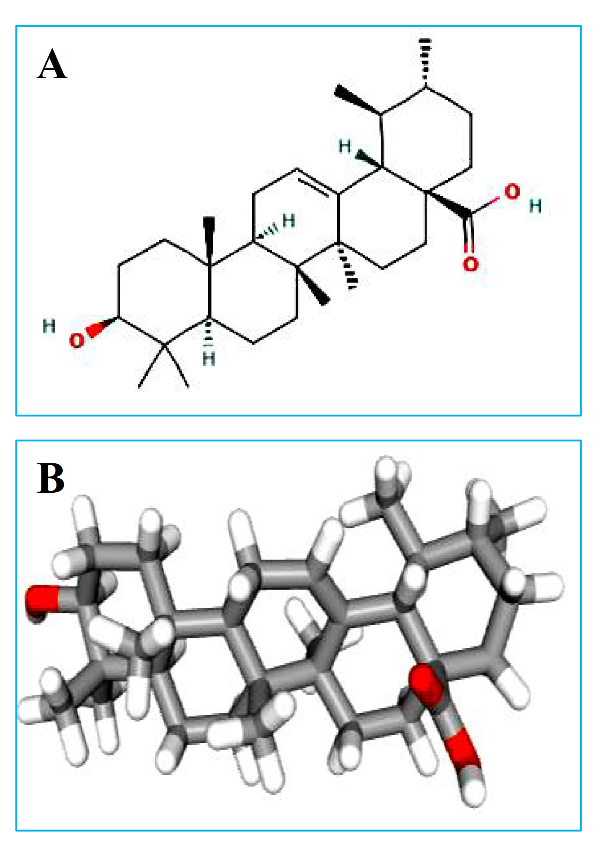


Supplementary Fig .1. Structure of UA. A is the 2D structure diagram of UA. B is the 3D structure diagram of UA.

Supplementary Fig.2


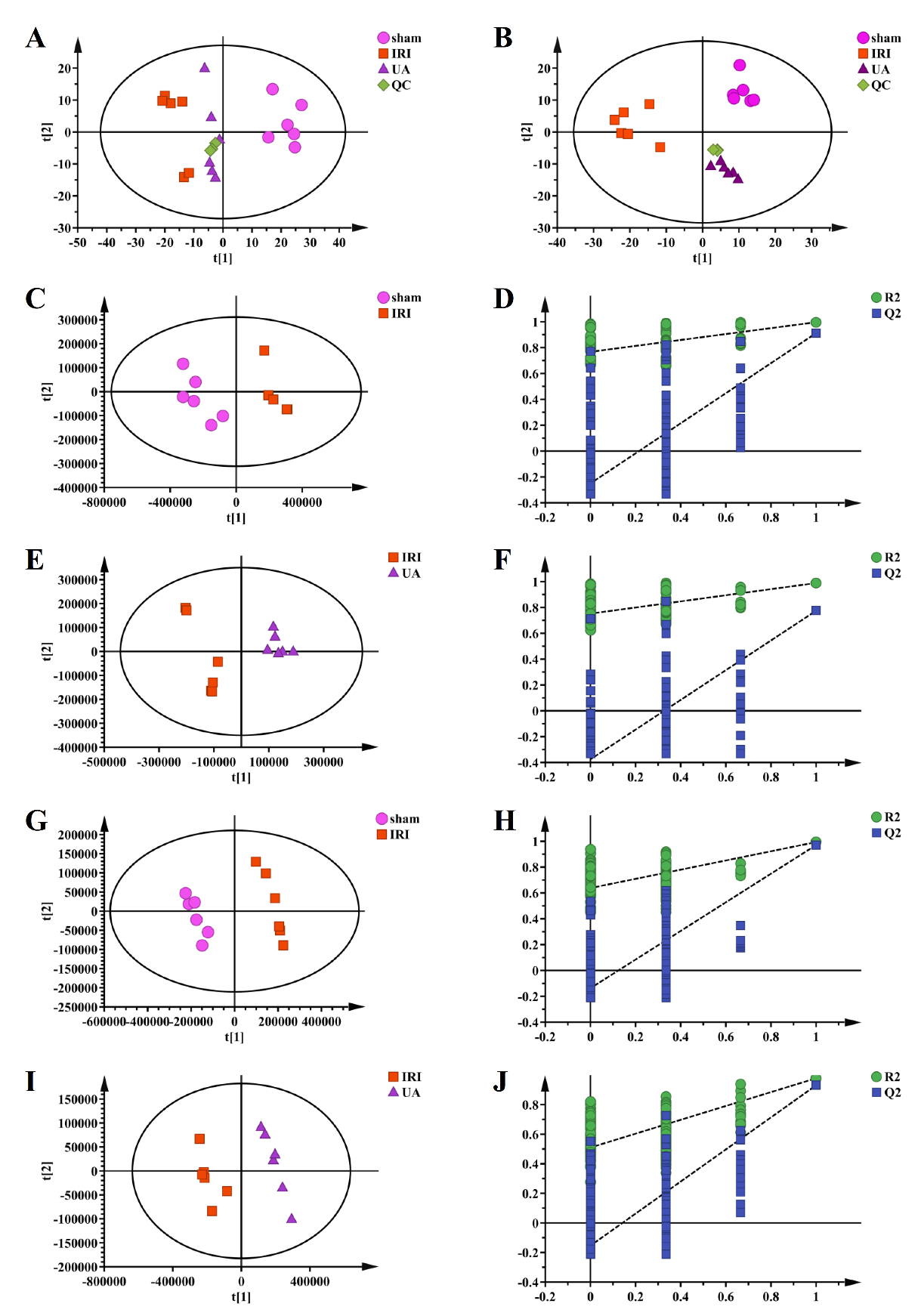


Supplementary Fig.2 Metabolomic analysis. A&B, Principal component analysis (PCA) in positive ion mode (A) and negative ion mode (B). C-J, squares-discriminant analysis (PLS-DA) between every two groups.

.

Supplementary Fig.3


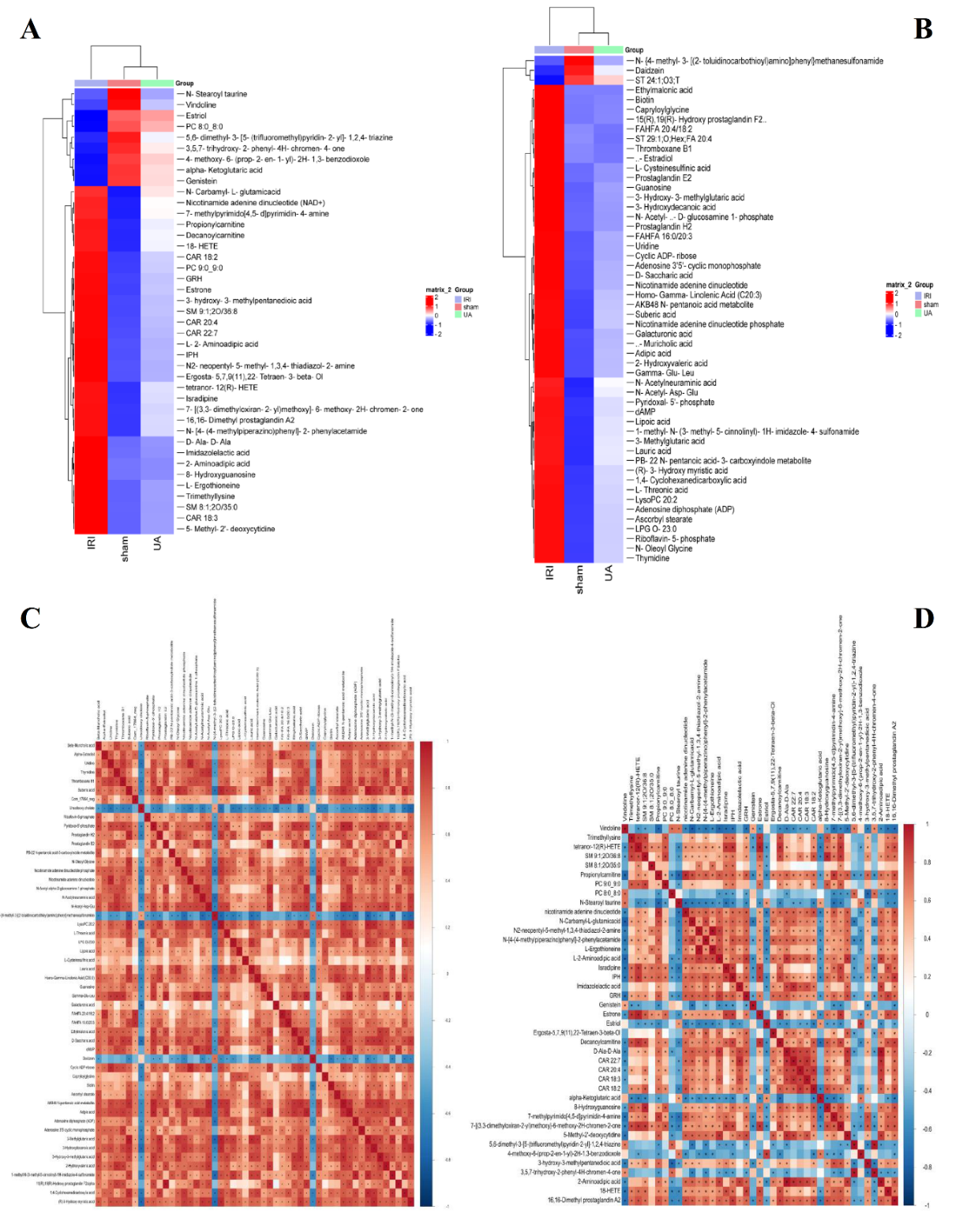


Supplementary Fig.3 Hierarchical clustering analysis (A-B) and Pearson correlation analysis (C-D).

Supplementary Fig 4


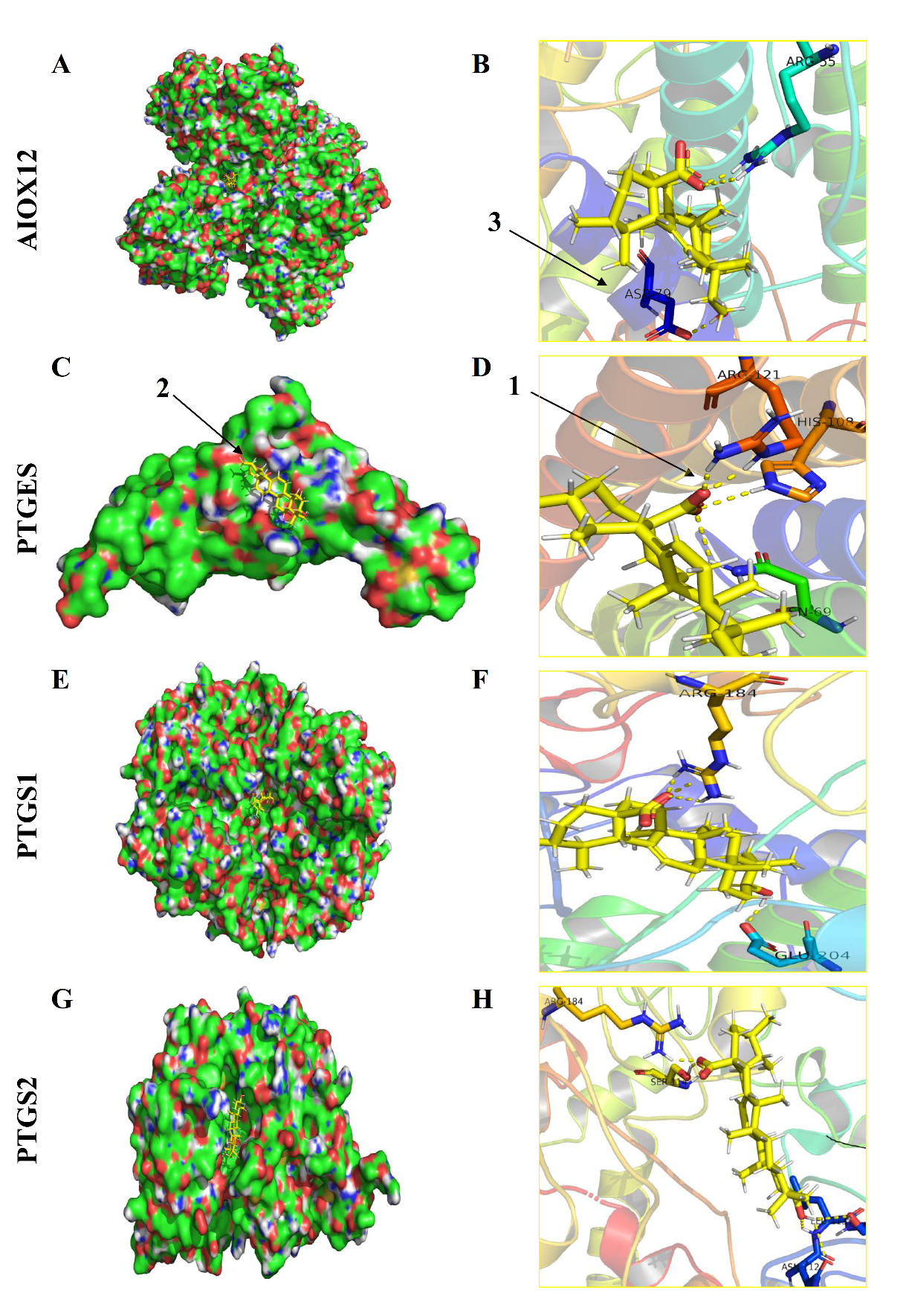


Supplementary Fig.4 Molecular docking diagram of ALOX12 (A), PTGES (B), PTGS1 (C) and PTGS2 (D). 1, 2, and 3 correspond to hydrogen bonds, small molecule compounds, and amino acid names, respectively.
